# Adolescent’s and youth’s adherence to antiretroviral therapy for better treatment outcome and its determinants: multi-center study in public health facilities

**DOI:** 10.1186/s12981-023-00588-y

**Published:** 2023-12-19

**Authors:** Yihenew Zurbachew, Desta Hiko, Girma Bacha, Hailu Merga

**Affiliations:** 1 AIDS Healthcare Foundation, Addis Ababa, Ethiopia; 2 Department of Epidemiology, Institute of Health, Jimma University, Jimma, Ethiopia; 3 Department of Nursing, Institute of Health, Jimma University, Jimma, Ethiopia

**Keywords:** Adherence, Antiretroviral therapy (ART), Youth, Adolescent, Determinants, Addis Ababa, Ethiopia

## Abstract

**Background:**

Low-adherence to Anti-retroviral therapy (ART) negatively affects the clinical, immunological, and virologic outcomes of patients. Adherence is the most important factor in determining Antiretroviral Therapy (ART) treatment success and long-term viral suppression which ultimately reduces morbidity and mortality. Thus, this study aimed to identify factors affecting adherence to antiretroviral therapy among adolescents and youth living with HIV.

**Methods:**

Facility-based cross-sectional study was conducted from March 21 to April 30, 2020 among 316 respondents in selected five high-loaded hospitals with adolescent and youth clients using systematic random sampling technique. Patients’ adherence was assessed when they had reportedly taken 95% or higher of their prescribed antiretroviral drugs in the five days before the interview. Data were collected, entered into EPI Data and exported to SPSS for analysis. Binary logistic regression was used to see the association between dependent and independent variables.

**Results:**

In this study, 316 respondents participated in the study, with a 99.7% response rate. The mean age of respondents were 17.94 years and majority of them (58.5%) were females. The overall ART adherence among adolescents and youths was found to be 70.6%. Being female (AOR = 0.323, 95% CI, 0.164–0.637), presence of opportunistic infection (AOR = 0.483, 95% CI, 0.249–0.936), taking additional medication beside ART (AOR = 0.436, 95% CI, 0.206–0.922) and availability of youth friendly services within the facility (AOR = 2.206, 95% CI, 1.031–4.721) were found to be predictors.

**Conclusion:**

The adherence rate in this study was low which is below the recommended adherence level. Being female, taking additional medication beside ART and presence of opportunistic infection were determinants of adherence. As a result, significant work must be done on opportunistic infection prevention through health education and promotion for screening and risk reduction. Similarly, adolescents and youths service integration with the ART Clinic is strongly advised.

## Background

Improving the duration and quality of life is the final goal of HIV care as well as treatment. To achieve this goal, medication adherence is mandatory. A benchmark of near-perfect adherence (≥ 95%) to antiretroviral therapy (ART) is often cited as necessary for HIV viral suppression [1, 2].

Effective Antiretroviral therapy (ART) leads to viral suppression, improves health, and prolongs the lives of adolescents and youths living with HIV/ AIDS; this decreases related morbidity and mortality [3, 4]. However, implementation of ART among Adolescents and youths 10–24 years faces major challenges in resource-limited settings [5]. HIV programs have gradually acknowledged teenagers as a crucial age group due to the high estimated rates of new infections among younger adolescents (15–19 years old) and the fact that many of the 920,000 of them getting antiretroviral medication (ART) survive into adolescence [6].

A systematic review and meta-analysis conducted globally on the adherence of antiretroviral therapy among adolescents living with HIV indicated that 62.3% of adolescents adhered to therapy. It indicated the lowest average ART adherence was in North America (53%), Europe (62%), and South America (63%), with higher levels in Africa (84%) and Asia [7].

In Ethiopia, it has been found that the prevalence of HIV among adults aged 15 to 59 is 0.9% with high prevalence in urban (2.9%) compared to rural (0.4%). This prevalence varies with sociodemographic and socio-economic factors. And women have twice the prevalence of HIV (1.2%) compared to men (0.6%) [8–10].

The focus of treatment has thereby changed from management of a severely debilitating disease to more long-term care with challenges such as maintaining treatment success, management of chronic co-morbidities, supporting adherence to life-long therapy, and prevention of HIV drug resistance [11–13].

Addis Ababa (the capital of Ethiopia) has the second highest prevalence next to the Gambella region. This suggests that there is a need to pay attention to factors affecting access to and outcomes of treatment among adolescents and youths. Adolescents and youth have higher rates of loss to follow-up (LTFU), poor adherence, and increased needs for psychosocial support and sexual reproductive health (SRH) services [4, 6]. Missing a prescribed regimen has a remarkable contribution to the emergence of treatment failure and resistance to HIV/AIDS drugs [14]. Moreover, only a few studies highlighted on adherence status of adolescents and youths living with HIV (AYPHIV), and available research in the country in general and in the study, area didn’t address factors affecting adherence status and their implication for intervention programs.

## Methods and materials

### Study design and setting

A facility-based cross-sectional study was conducted from March 21–April 30, 2020, in selected public hospitals in Addis Ababa, the Ethiopian capital. Those selected hospitals were Alert Hospital, Tikur Anbesa Specialized Hospital, Yekatit 12 referral Hospital, Zewditu General Hospital, and St. Paul Specialized Hospital. The city has one city council, 10 sub-cities, and 117 woredas with 4.5 million. Under its administration, there are six public hospitals, one public health laboratory, and two health science colleges. Ten sub-city health offices are directly accountable to their respective sub-city administrations. A total of 7826 adolescents and youths living with HIV, ages ranging from 10 to 24 years attend ART clinics in the city.

### Population and sampling

All selected adolescents and youths living with HIV aged 10 to 24 years attending ART clinics in selected public hospitals in Addis Abeba City administration were the study population. HIV-positive adolescent and youth (10–24 years) clients who have followed ART clinics at least for six months were included, whereas those who were seriously ill and unable to respond during data collection were excluded from the study. The sample size for this study was calculated using a double population proportion formula with the following assumptions: 95% confidence level, 80% power, and 86% adherence to co-trimoxazole preventive therapy as an exposure variable from a previous study [15]. Finally, with the addition of a 10% non-response rate, the final sample size became 317 and each size was proportionally allocated to each five selected hospitals. The study was conducted in the aforementioned selected five loaded high-loaded hospitals with adolescent and youth clients greater than 150. The total sample size was proportionally allocated with the number of Adolescents and youth on follow-up using a systematic random sampling technique.

### Measurements

#### Adherence to ART

In this study, patients had adherence to ART when they had reportedly taken 95% or higher of their prescribed antiretroviral drugs in the five days before the interview. High-level ART adherence is said if the clients take ≥ 95% of the prescribed drug properly as prescribed by the health care provider within the last week before the date of the interview. Poor adherence was said if the clients take < 95% of the prescribed drug properly as prescribed by the health care provider within the last week before the date of the interview [5, 7, 15].

### Data collection tool and procedure

A pre-tested structured interviewer-administered questionnaire which was pre-tested was used for data collection. The data collection tool was developed after reviewing relevant literature [5, 7, 8, 11, 15]. The questionnaire contained variables related to socio-demographic characteristics, patient and family caregiver-related factors, medication-related factors, and health care delivery systems-related factors. Five youth adherence supporters collected data and two supervisors supervised it. To maintain the data quality, data collectors and supervisors received a one-day training on the purpose and procedure of data collection related to this research. Data were checked for completeness and consistency after each day of data collection. Moreover, a pretest was done on 5% of the total sample size at a standalone AIDS Healthcare foundation ART clinic located in Addis Ababa. Then, few modifications were done on the concept and order of few questions.

### Data analysis

Data were analyzed and reported according to STROBE recommendations [16]. The collected data were entered into Epi Data version 3.1 software and exported to the statistical package for social studies (SPSS) version 25.0 software for analysis. Descriptive analysis was done. Binary logistic regression was carried out to see the association of each of the independent variables with the outcome variable. For variables having a *p*-value less than 0.25, the multivariable logistic regression method was used to identify factors associated with adherence. The results were claimed to be statistically significant when the *p*-value was < 0.05 at a 95% confidence level. Multicollinearity and model fitness were checked with VIF and Hosmer and Lameshow goodness of fit test model respectively.

## Results

### Socio-demographic characteristics of respondents

Three hundred sixteen respondents participated in the study, with a 99.7% response rate. The mean age (SD) of respondents were 17.94 (2.84 SD). More than half, 185 (58.5%) of them were females and 65.5% of them were orthodox by their religion. Two hundred thirty-three (73.7%) of them attended secondary school and above in educational status. (Table 1).

**Table 1:** Socio-demographic and socio-economic characteristics of respondents on Magnitude and factors associated with adherence among Adolescents and youth, Addis Ababa, Ethiopia, 2020 (n = 316)

Variable	Variable category	Frequency (%)
Sex	Male Female	131 (41.5%) 185 (58.5%)
Age	Mean (SD)	17.94 (2.84)
Religion	Orthodox Muslim Protestant Catholic Others	207 (65.5%) 36 (11.4%) 61 (19.3%) 7 (2.2%) 5 (1.6%)
Educational status	Illiterate Primary Secondary and above	3 (1.0%) 79 (25.0%) 233 (73.7%)
Occupational status of family	Merchant Private organization employee Government employee Driver Daily laborer Self-employed	51 (16.4%) 50 (16.1%) 82 (26.4%) 29 (9.2%) 29 (9.2%) 70 (22.5%)
Marital status of parents	Married Never Married Separated Divorced Widowed	192 (61.3%) 18 (5.8%) 10 (3.2%) 37 (11.8%) 56 (17.9%)
Living situation	With parents With relative/s Alone	217 (68.9%) 84 (26.7%) 14 (4.4%)
Family monthly income	Mean (SD)	6304.5 (7170.7)
Family size	Mean (SD)	4.2 (1.7)
Respondent`s monthly income	Mean (SD)	2388.5 (1738.8)

### Respondents’ clinical information and drug adherence

The mean age of the participants at disclosure who acquired the infection through vertical transmission was 11.90 ± 2.78 SD. Knowing the WHO Clinical Staging system has been shown to be a practical and accurate way to manage HIV-infected patients. Accordingly, one hundred seven (34.3%) were at WHO stage 2 at the time of ART initiation and the majority 197 (62.3%) were on first-line treatment. More than two-thirds (38.6%) of the participants responded that they faced depression or anxiety and two hundred fifty-nine (82.0%) of the respondents had emotional support. Two hundred twenty (69.6%) of them had a family member living with HIV and about nine-tenths (90.5%) had support from family. One hundred (31.8%) of respondents had a history of opportunistic infection.

Two hundred nineteen participants (69.3%) received 30 doses of the prescribed drug per month. In this study, some participants missed some doses for the last months before the data collection time. Accordingly, 10.4%, 13.0%, and 23.7% missed their prescribed doses in a day, three days ago, and four days before data collection time respectively. This might be due to forgetting and feeling sick or uncomfortable because of the drug. The overall adherence level (operationally defined under measurement section above) was 233 (70.6%) with (95% CI: 66.0-76.0%), measured by taking the number of doses missed within 5 days. If no dose was missed within the past five days, it was considered as adhered. Two hundred sixty-four (83.5%) of the respondents had adherence counseling services within the facility. The majority of the respondents responded that they had youth programs at the health facilities 285 (90.2%) and services available for their concerns 275 (87.0%) in the health facilities.

### Determinants of adherence to antiretroviral therapy

The result in multivariable logistic regression revealed the sex of respondents, history of opportunistic infection, and having additional medication and service for your concern were significantly associated with ARV adherence. Female respondents were 68% less likely to adhere to ARV as compared to their counterparts (AOR = 0.323, 95% CI, 0.164–0.637). Those who had a previous history of opportunistic infection and taking additional medications were 52% and 56% less likely to adhere (AOR = 0.483, 95% CI, 0.249–0.936) and (AOR = 0.436, 95% CI, 0.206–0.922) respectively. Those who had service for their concern within the facility were more than two times to adhere as compared to their counterpart (AOR = 2.206, 95% CI, 1.031–4.721) (Table 2).

**Table 2:** Multivariate analysis on the magnitude and factors associated with adherence among adolescents and youths, Addis Ababa, Ethiopia, 2020 (n = 316)

Variables		Adhered	Non-Adhered	COR (95%)	AOR (95%)	P value
Sex	Male Female	103 (78.6%) 120 (64.3%)	28 (21.4%) 65 (35.1%)	1 0.502 (0.300–0.840)	1 0.323 (0.164–0.637) *	0.001*
WHO stage at start of ART	Stage 1 Stage 2 Stage 3 Stage 4	82 (79.6%) 70 (71.4%) 56 (67.5%) 15 (46.9%)	21 (20.4%) 28 (28.6%) 27 (32.5%) 17 (53.1%)	1 0.640 (0.334–1.226) 0.531 (0.273–1.032) 0.226 (0.097–0.525)	1 1.117 (0.480–2.598) 0.881 (0.374–2.073) 0.386 (0.142–1.052)	0.797 0.771 0.063
History of opportunistic Infection	Yes	57 (57.0%)	43 (43.0%)	0.394 (0.237–0.654)	0.483 (0.249–0.936) *	0.031*
Additional medication	Yes	31 (47.7%)	34 (52.3%)	0.280 (0.159–0.494)	0.436 (0.206–0.922) *	0.030*
Availability of service in the facility	Yes	68 (75.6%)	22 (24.4%)	1.416 (0.811–2.471)	2.206 (1.031–4.721) *	0.042*

Note: 1 reference categories * statistically significant at p value = 0.05

## Discussion

In this study, the overall adherence rate was 70.6% which is lower than the recommended adherence level of at least 95% to fully benefit from ART [17]. Similarly, it is lower than a study conducted among American adolescents aged 12–18 years revealed low ART adherence level [18]. A study done in Botswana reported that a high proportion (76.9%) of the studied adolescents had excellent ART adherence though it was done in a single health facility [19]. A systematic review of 50 articles involving 10,725 adolescents on ART reported an overall adherence level to be 62.3%. In this study, the ART adherence level for Africa was found to be 84% which is higher than the current study’s 70.6% [7]. A study conducted in Addis Ababa among adolescents aged rage 13–19 at three hospitals revealed a high adherence level (79.1%) [15]. This difference might be due to the sample size difference and the current study was done in the age range of 10–24 years. Contrary to these findings, a study that measured adherence using unannounced home-based pill count revealed a very low adherence level, 34.8%. This study was done among HIV-infected children below 15 years who were attending the pediatric ART clinic of Tikur Anbessa Hospital in Addis Ababa [20].

The sex of the respondents had a positive association with adherence to ARVs. Male respondents were more adherent to ARV as compared to their female counterparts which is similar to studies done in Ambo, central Ethiopia [21], India [22] and Uganda [23]. This might be due to forgetfulness, poor communication and the side effects of drugs. Furthermore, due to cultural barriers, women in this study area may have had difficulties disclosing their status, thus meaning that they need to hide while taking their medications. On the other hand, this study is not in line with a study done in Zambia in which male adolescents were found to be two times more non-adherent than female adolescents [24].

Respondents who had a previous history of opportunistic infections were less likely to adhere as compared to those who didn’t. This is in line with a study done in sub-Saharan Africa [25] but in contrast with finding from Uganda [23]. This might be due to the patient who had an opportunistic infection was forced to take other drugs. This might lead to pills burden which imposed non-adherence or a patient who had opportunistic infection might to lead bedridden which might force them to discontinue taking ART drugs.

The study shows that those who took additional medications along with ART were less likely to adhere to their ART compared to those who didn’t have additional medication. This finding is in agreement with the study done in southern Ethiopia [26]. However, the study conducted in Addis Ababa described that those who took co-trimoxazole preventive therapy (CPT) besides ART were more than 3 times more likely to adhere than those who didn’t [15]. The reason for non-adherence while taking additional medication might be due to pill burden drug interaction and the possibility of side effects. This finding also indicated that those adolescents and youths who got service for their concerns within the health facility were two times more adhered to than their counterparts. This might be due to the satisfaction with the service provided for them in the health facility.

This study has some limitations. First, adherence assessment was based on patients’ self-report which might affect the estimation effect. Second, the study considered anyone who have ever missed their dose as non-adherent regardless of number of missed dose and time since missed. Despite attempts taken during data collection, the study may be affected by social desirability and recall biases.

## Conclusions

The findings of this study indicated low ART adherence rate among adolescents and youths. Gender, presence of opportunistic infection, taking additional medication besides ART, and service for concerns within the facility were identified as significant predictors of non-adherence. Thus, emphasis should be given to early treatment of opportunistic infections and adolescent and youth service integration with ART clinics to strengthen those population groups to adhere to their ART therapy.

## Data Availability

The datasets used or analyzed during the current study are available from the corresponding author on reasonable request.
